# A Novel Coagulation Classification and Postoperative Bleeding in Severe Spontaneous Intracerebral Hemorrhage Patients on Antiplatelet Therapy

**DOI:** 10.3389/fnagi.2022.793129

**Published:** 2022-02-16

**Authors:** Qingyuan Liu, Xiong Li, Nuochuan Wang, Junhua Yang, Kaiwen Wang, Shanwen Chen, Jiangan Li, Jun Wu, Yanan Zhang, Shuo Wang

**Affiliations:** ^1^Department of Neurosurgery, Beijing Tiantan Hospital, Capital Medical University, Beijing, China; ^2^China National Clinical Research Center for Neurological Diseases, Beijing, China; ^3^Department of Neurosurgery, Beijing Chaoyang Hospital, Capital Medical University, Beijing, China; ^4^Department of Blood Transfusion, Beijing Tiantan Hospital, Capital Medical University, Beijing, China; ^5^Department of Neurosurgery, Second People's Hospital of Wuxi City, Nanjing Medical University, Wuxi, China

**Keywords:** severe spontaneous intracerebral hemorrhage, antiplatelet therapy (APT), coagulation classification, thrombelastography, postoperative rebleeding

## Abstract

**Background and Purpose:**

For patients with severe spontaneous intracerebral hemorrhage on antiplatelet therapy (patients with APT-SICH), postoperative rebleeding (PR) is an important cause of poor outcomes after surgery. As impacted by coagulation disorder caused by APT, patients with APT-SICH are likely to suffer from PR. This study aimed to assess the risk of PR in patients with APT-SICH receiving emergency surgery using a novel coagulation classification.

**Methods:**

This prospective, multicenter cohort study consecutively selected patients with APT-SICH between September 2019 and March 2021. The preoperative coagulation factor function was recorded, and the platelet function was assessed using thrombelastography. Based on platelet and coagulation factor function, a novel four-type coagulation classification, i.e., Type I (severe coagulation disorder), Type IIa (low platelet reserve capacity), Type IIb (normal coagulation), and Type III (hypercoagulation), was presented. The primary outcome was PR, defined as the rebleeding in the operative region or new intracerebral hemorrhage correlated with the operation.

**Results:**

Of the included 197 patients with APT-SICH, PR occurred in 40 patients (20.3%). The novel coagulation classification categorized 28, 32, 122, and 15 patients into Type I, Type IIa, Type IIb, and Type III, respectively. The Type I patients had the highest incident rate of PR (39.3 per 100 persons), followed by the Type IIa patients (31.3 per 100 persons). In the PR-related analysis, the large hematoma volume (hazard ratio (HR): 1.02; 95% CI: 1.02–1.03; *p* < 0.001), Type I (HR: 9.72; 95% CI: 1.19–79.67; *p* = 0.034), and Type IIa (HR: 8.70; 95% CI: 1.09–69.61; *p* = 0.041) were correlated with the highest risk of PR. The coagulation classification could discriminate the PR patients from no PR (NPR) patients (*p* < 0.001), and it outperformed the conventional coagulation assessment (only considering platelet count and coagulation factor function) (c-statistic, 0.72 vs. 0.55).

**Conclusion:**

The novel coagulation classification could discriminate the patients with APT-SICH with the highest risk of PR preoperatively. For the Type I and Type IIa patients, emergency surgery should be performed carefully.

## Introduction

Spontaneous intracerebral hemorrhage (SICH) is the acute subtype of stroke, characterized by high morbidity and mortality (Cordonnier et al., 2018). Severe SICH is life-threatening because of rapidly increasing intracranial pressure and subsequent cerebral hernia (Hemphill et al., 2015; Kim and Bae, 2017; Cordonnier et al., 2018). As reported previously, emergency surgery can improve the outcome of SICH by reducing mortality (Hemphill et al., 2015; Bhaskar et al., 2017; Lo et al., 2017; Cai et al., 2019; Luzzi et al., 2019; Wu et al., 2021a). However, for patients on continuous antiplatelet therapy (APT), the coagulation dysfunction caused by APT could increase the risk of hemorrhagic complications after emergency surgery. Postoperative rebleeding (PR) is a notable complication for these patients after surgery, which can cause a catastrophic outcome. Our previous study indicated that the incident rate of PR was 21.9% in patients with SICH on APT (patients with APT-SICH), which was approximately two times as much as patients without a history of APT (Wu et al., 2021a).

Coagulation is important to ensure the safety of surgery. The APT can inhibit the platelet function, which is essential in coagulation. As impacted by the potential coagulation dysfunction caused by APT, most guidelines and consensus recommended that surgery is not considered until the coagulation function recovers (Baron et al., 2013; Khoo and Lepas, 2014; Hornor et al., 2018). However, our previous study revealed that emergency surgery is still safe for a considered part of patients with APT-SICH (Wu et al., 2021a). At present, fewer tools or methods are available for assessing the platelet function in patients with APT-SICH who need emergency surgery. Notably, existing preoperative examinations only screen the coagulation factor dysfunction, instead of the platelet dysfunction. Thus, the risk of surgery for patients with APT-SICH cannot be reflected.

This prospective, multicenter cohort study aimed to build a novel coagulation classification by combining the function of platelet and coagulation factor and further assess its clinical utility to evaluate the risk of PR in patients with APT-SICH.

## Methods

### Standard Protocol Approvals, Registrations, and Patient Consents

Surgical Treatments for Antiplatelet Intracerebral Hemorrhage (SAP-ICH) Study (unique identifier: ChiCTR1900024406) refers to a prospective, multicenter cohort study. This study was approved by institutional ethics committees. All patients (or guardians of patients) provided written informed consents. The protocol of SAP-ICH study was published previously (Wu et al., 2021a).

### Study Design and Subjects

The present prospective cohort study was a preliminary analysis of SAP-ICH cohort and consecutively selected eligible patients with APT-SICH from 14 hospitals (six regional medical centers and eight collaborative hospitals of these medical centers) between September 2019 and March 2021. Preoperative “on the APT” was defined as patients who underwent a long-term (more than 7 days) oral APT and discontinued APT within 7 days after SICH. In this study, antiplatelet medication included aspirin only (100 mg/day), clopidogrel only (75 mg/day), and aspirin + clopidogrel [dual antiplatelet therapy (DAPT)].

**Figure 1:**
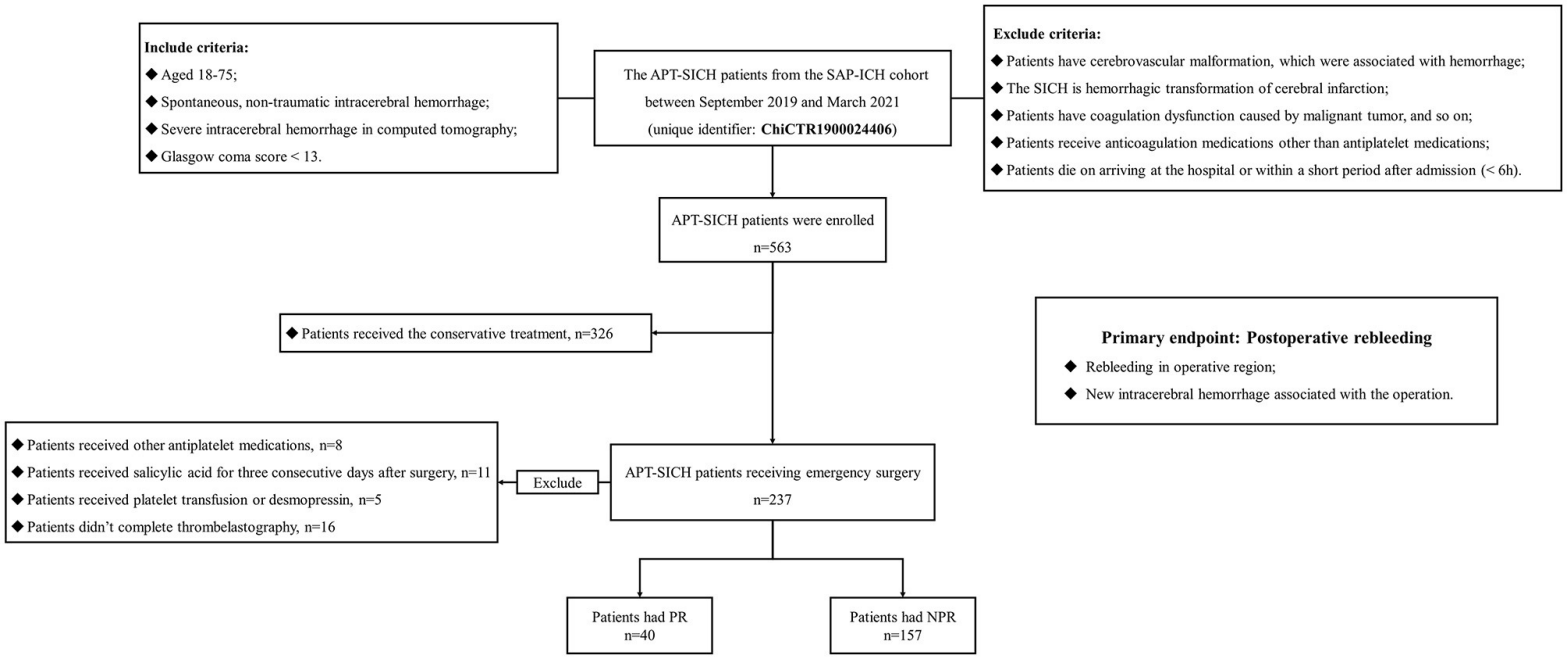
The flowchart of patient enrollment. A total of 563 eligible APT-SICH were enrolled. Of them, 221 patients received emergency surgery. We further excluded 8 patients taking other antiplatelet medications, 11 patients receiving salicylic acid for three consecutive days after surgery, five patients receiving transfusion or desmopressin, and 16 patients without thrombelastography. Finally, 197 patients were included in this study. APT-SICH, patients with severe spontaneous intracerebral hemorrhage on antiplatelet therapy.

As shown in Figure 1, patients with APT-SICH were selected under the following criteria: inclusive criteria: (1) patients aged 18–75 years; (2) patients having spontaneous, nontraumatic intracerebral hemorrhage; (3) patients having radiological SICH, which is defined as supratentorial hematoma volume > 30 ml, infratentorial hematoma volume > 10 ml, midline shift > 1 cm, or large intraventricular hematoma, in computed tomography (CT); (4) patients having a Glasgow Coma Score (GCS) <13, and exclusive criteria: (1) patients having cerebrovascular malformation (e.g., arteriovenous malformation or intracranial tumor), correlated with hemorrhage; (2) the SICH referring to hemorrhagic transformation of cerebral infarction; (3) patients having coagulation dysfunction attributed to malignant tumor; (4) patients receiving anticoagulation medications other than antiplatelet medications; and (5) patients died on arriving at the hospital or within a short period after admission (<6 h). Of all the selected patients with APT-SICH, 221 patients underwent emergency surgery. We further excluded (1) eight patients receiving other antiplatelet medications, i.e., dipyridamole, tirofiban, and ticagrelor; (2) 11 patients received salicylic acid, e.g., aspirin effervescent tablets and aspirin-dl-lysine, for three consecutive days after surgery; (3) five patients received platelet transfusion or desmopressin, which could reverse the platelet function; and (4) 16 patients not undergoing thrombelastography (TEG) test. Finally, 197 patients with APT-SICH undergoing emergency surgery were included in this study.

### Data Collection

The demographic information (i.e., sex, age, history of smoking, and alcohol consumption), comorbidities (i.e., dyslipidemia, diabetes mellitus, coronary heart disease, ischemic stroke, and history of intracerebral hemorrhage), neurological status on admission [i.e., GCS, modified Rankin scale (mRS)], and laboratory examination [i.e., platelet count, partial thromboplastin time (APTT), prothrombin time (PT), and fibrinogen (Fbg)] were collected from electronic medical records. The medication used for treating prehemorrhage (i.e., dosage, frequency, and duration) was recorded by an investigator by consulting the family members or guardians of patients. Radiological features were examined by two investigators (X. L. and K.W., blind to patient information) based on the CTs performed before surgery. The discrepancies were solved by consulting a senior neurosurgeon (J.W.). To be specific, the features consisted of the left side, hemorrhage location, bleeding to the ventricle, hematoma volume, and preoperative hematoma extension. The hematoma volume was semiautomatically measured with the 3-dimensional geometry supported by the Brainlab workstation based on the preoperative CT. The ICH score was calculated by complying with the age, GCS, hematoma volume, and hemorrhage location (Hemphill et al., 2001). Regarding alcohol consumption, the included patients were classified as regular alcohol abuse (drinking once or more per week) and others (Cochrane et al., 2003). Given the smoking condition, patients were classified into current smoker and others (Can et al., 2017).

For TEG, the parameters correlated with the platelet function were mainly collected, including citric acid kaolin tracing (CK)-maximum amplitude (MA), the inhibition caused by aspirin (AA%), and the inhibition attributed to clopidogrel (ADP%). An experienced technicist (N.W.) would conduct the quality control of the TEG.

### Preoperative Coagulation Assessment

In accordance with the recommendation of coagulation for surgery (Spahn et al., 2019), this study defined patients with any one of APTT prolonged by ≥ 10 s (APTT ↑), PT prolonged by ≥ 3 s (PT ↑), or Fbg <1.5 g/L (Fbg ↓) as coagulation factor dysfunction.

**Figure 2:**
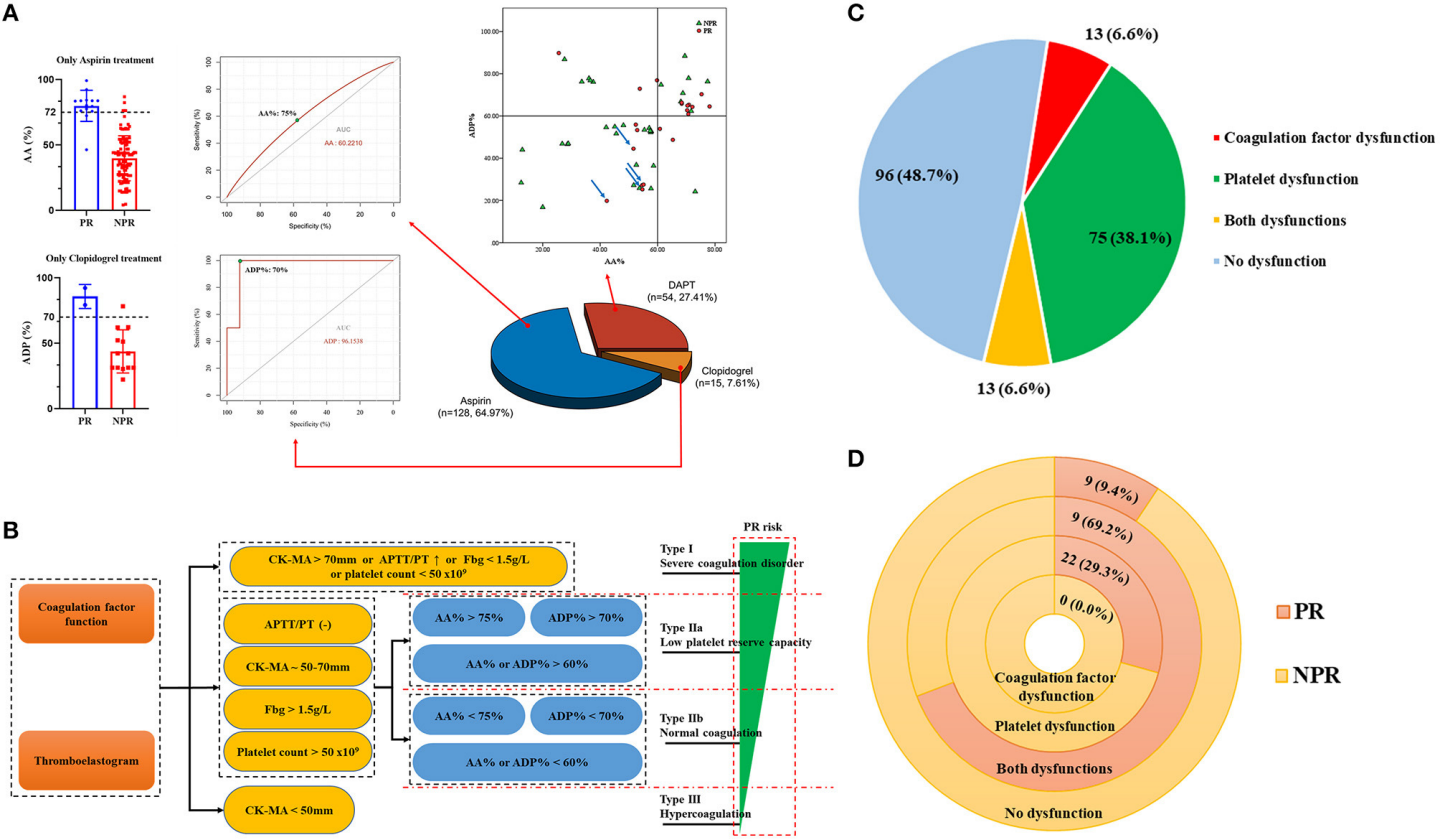
Preoperative platelet function and novel coagulation classification. **(A)** The AA% at 75% for patients receiving aspirin only, the ADP% at 70% for patients receiving clopidogrel only, the AA%/ADP% at 60% for patients receiving DAPT could discriminate patients with PR from patients with NPR. **(B)** This classification categorized all patients into four types: Type I, patients having platelet count ↓, platelet hypofunction, or coagulation factor dysfunction; Type IIa, patients having normal platelet count, normal platelet and coagulation factor function, and AA%/ADP% ↑; Type IIb, patients having normal platelet count, normal platelet and coagulation factor function, and no AA%/ADP% ↑; Type III, patients having platelet hyperfunction. **(C)** The characteristics of preoperative coagulation disorder. **(D)** The incident rate of PR in different coagulation disorders. PR, postoperative rebleeding; NPR, no postoperative rebleeding; DAPT, dual antiplatelet therapy; AA%, the inhibition caused by aspirin; ADP%, the inhibition caused by clopidogrel; CK-MA, citric acid kaolin tracing-maximum amplitude.

The platelet count <50 × 10^9^ was recognized as platelet count ↓. As for TEG, the CK-MA <50 mm was considered as the platelet hyperfunction, and CK-MA > 70 mm was considered as the platelet hypofunction. As shown in Figure 2A, increased AA% and ADP%, which could reflect the low platelet reserve capacity, were also correlated with PR. Given the Youden index of AA% and ADP%, the AA% at 75% for patients receiving aspirin only, the ADP% at 70% for patients receiving clopidogrel only, and the AA% or ADP% at 60% for patients receiving DAPT could discriminate patients with PR from patients without PR. The AA% or ADP% over than the mentioned cutoff values in the corresponding conditions was identified as AA% ↑ or ADP% ↑. Notably, for patients with CK-MA <50 mm or > 70 mm, the AA% and ADP% could be meaningless; hence, the AA% and ADP% were only considered when CK-MA was 50–70 mm (the mind mapping presented in Supplementary Figure 1). The patients with any one of platelet count ↓, platelet hypofunction (CK-MA > 70 mm), or normal CK-MA but AA%/ADP% ↑ were classified as platelet dysfunction.

As shown in Figure 2B and Supplementary Table 1, based on the platelet and coagulation factor function, we categorized all patients into four types: Type I, patients with severe coagulation disorder, defined as platelet count ↓, platelet hypofunction (CK-MA > 70 mm), or coagulation factor dysfunction (APTT ↑, PT ↑, or Fbg ↓); Type II, the platelet function (CK-MA~50–70 mm) and coagulation factor function were normal, but if the AA% > 75% for patients with aspirin only, ADP% > 70% for patients with clopidogrel only, or AA%/ADP% > 60% for patients with DAPT was recognized as Type IIa (low platelet reserve capacity), otherwise as Type IIb (normal coagulation); Type III, patients with hypercoagulation (CK-MA <50 mm). For no patient with hyperfunction of the coagulation factor in this cohort, the hypercoagulation was usually the platelet hyperfunction.

The conventional coagulation assessment only considers platelet count and coagulation factor function. Thus, patients with any one of platelet count ↓, APTT ↑, PT ↑, or Fbg ↓ were recognized as the conventional coagulation disorder.

### Outcome

The primary endpoint was the PR. All patients were observed until they were discharged. CTs would be routinely performed at 4, 24, and 72 h after surgery. If a patient showed the signs of deteriorating neurological status, one or more CTs would be performed to identify whether there was PR. The interval from the end of emergency surgery to PR or discharge was calculated.

The PR was identified when the radiological examination identified a rebleeding in the operative region or new intracerebral hemorrhage correlated with the operation on radiological examination (compared with the preoperative CT or last CT), new subdural hemorrhage, new subarachnoid hemorrhage, or new intraventricular hemorrhage; otherwise, it was considered as no PR (NPR). Two experienced neurosurgeons (JH. Y. and SW. C.), blind to the information of patients, identified this event based on the postoperative CTs and categorized all patients into PR group and NPR group. The discrepancies were solved through the consultation with a senior neurosurgeon (S.W.).

### Statistical Analysis

Categorical variables were presented as numbers (no.) and percentages (%). Continuous variables with normal distribution were expressed as means and standard deviation, and medians and interquartile range (IQR), if otherwise. The differences between the groups in continuous variables were compared by performing the Student's *t*-tests or Wilcoxon rank-sum tests, and the differences in categorical variables were compared using the chi-square tests or Fisher's exact tests. The incident rate (IR) of PR and its 95% confidence interval (CI) were calculated. The univariate and multivariate Cox regression analyses were performed in PR-related analysis. The result of Cox regression analysis was presented as hazard ratio (HR) and 95% CI. The receptor receiver curve (ROC) analysis was performed to test the predictive accuracy of different coagulation assessment methods for the PR using the area under the curve (AUC). A model with AUC > 0.7 and *p* < 0.05 was considered to be the clinical utility. The statistical analyses were conducted using SPSS 24.0 (SPSS, Chicago, IL), considering the two-sided *p* < 0.05 statistically significant.

## Results

### Demographic and Clinical Features

Of the included 197 patients with APT-SICH undergoing emergency surgery, PR occurred in 40 patients (20.3%). Demographic and clinical information are listed in Table 1. More patients with PR had an intracerebral hemorrhage history (27.5 vs. 9.6%, *p* = 0.003). Of the included patients, 117 (59.4%) patients received craniectomy, 24 (12.2%) received endoscopic surgery, and 56 (28.4%) received minimally invasive surgery; no significance was reported in the PR rate of different surgeries (*p* = 0.084). As for the APT history, the patients receiving DAPT had the highest rate of PR, followed by patients receiving aspirin or clopidogrel (*p* < 0.001). The median hematoma volume of patients with PR was higher than that of patients with NPR (85.9 vs. 44.8 ml, *p* < 0.001). The patients with PR had lower Fbg level than patients with NPR (2.6 vs. 2.8 g/L, *p* = 0.004). The median time from hemorrhage to surgery was 18.3 (13.4–32.2) h for patients with PR and 25.6 (15.0–36.5) h for patients with NPR; however, the difference was not statistically significant (*p* = 0.077).

**Table 1:** The information of included patients with APT-SICH.

**Characteristics**	**PR ***n*** = 40**	**NPR ***n*** = 157**	***P*** **value**
Male -no. (%)	31 (77.5%)	116 (73.9%)	0.640
Age -median (IQR)-years	63 (51–72)	56 (48–66)	0.155
Comorbidities			
Dyslipidemia -no. (%)	3 (7.5%)	14 (8.9%)	0.776
Diabetes mellitus -no. (%)	12 (30.0%)	42 (26.8%)	0.682
Coronary heart disease -no. (%)	12 (30.0%)	46 (29.3%)	0.931
Ischemic stroke -no. (%)	17 (42.5%)	43 (27.4%)	0.064
Intracerebral hemorrhage history -no. (%)	11 (27.5%)	15 (9.6%)	0.003^+^
Current smoker -no. (%)	18 (45.0%)	48 (30.6%)	0.085
Regular alcohol abuse -no. (%)	4 (10.0%)	18 (11.5%)	0.793
Antiplatelet therapy prehemorrhage -no. (%)			<0.001^+^
Aspirin	17 (42.5%)	111 (70.7%)	
Clopidogrel	2 (5.0%)	13 (8.3%)	
DAPT	21 (52.5%)	33 (21.0%)	
Clinical/CT findings on admission			
Left side -no. (%)	14 (35.0%)	51 (32.5%)	0.345
Hemorrhage location -no. (%)			0.181
Supratentorial deep	19 (47.5%)	102 (65.0%)	
Supratentorial lobar	19 (47.5%)	33 (21.0%)	
Cerebella	2 (5.0%)	22 (14.0%)	
Bleeding to ventricle -no. (%)	23 (57.5%)	107 (68.2%)	0.205
Hematoma volume -median (IQR)-cc	85.9 (81.4–95.0)	44.8 (33.2–69.7)	<0.001^+^
Preoperative hematoma extension -no. (%)	10 (25.0%)	41(26.1%)	0.886
Neurological statues on admission			
mRS -median (IQR)	4 (4–5)	4 (4–5)	0.379
GCS -median (IQR)	5.5 (3–9)	6 (3–9)	0.659
ICH score -median (IQR)	3 (3–4)	3 (2–3)	0.088
Surgery-no. (%)			0.084
Craniectomy	27 (67.5%)	90 (57.3%)	
Endoscopic surgery	5 (12.5%)	19 (12.1%)	
Minimally invasive surgery^†^	8 (20.0%)	48 (30.6%)	
Time from hemorrhage to surgery -median (IQR)-hours	18.3 (13.4–32.2)	25.6 (15.0–36.5)	0.077
Preoperative laboratory examination			
Platelet count -median (IQR)- × 10^9^	216.0 (199.5–226.8)	216.3 (206.0–238.7)	0.338
APTT -median (IQR)-s	24.8 (22.4–28.0)	24.6 (24.5–30.4)	0.078
PT -median (IQR)-s	12.6 (12.0–13.1)	13.4 (12.3–13.1)	0.264
Fbg -median (IQR)-g/l	2.6 (2.2–3.1)	2.8 (2.4–3.6)	0.004^+^
CK-MA -no. (%)			<0.001^+^
>70 mm	11 (27.5%)	7 (4.5%)	
50~70 mm	28 (70.0%)	136 (86.6%)	
<50 mm	1 (2.5%)	14 (8.9%)	
Novel coagulation classification			<0.001^+^
I	11 (27.5%)	17 (10.8%)	
IIa	10 (25.0%)	22 (14.0%)	
IIb	18 (45.0%)	104 (66.3%)	
III	1 (2.5%)	14 (8.9%)	

+ *The parameter is statistically significant*.

† *Minimally invasive surgery included the only minimally invasive surgery and minimally invasive surgery + fibrinolytic therapy*.

*APT-SICH, severe spontaneous intracerebral hemorrhage patients on antiplatelet therapy; PR, postoperative rebleeding; NPR, no postoperative rebleeding; DAPT, dual antiplatelet therapy; mRS, modified Rankin scale; GCS, Glasgow Coma Score; APTT, activated partial thromboplastin time; PT, prothrombin time; Fbg, fibrinogen*.

### Preoperative Coagulation Disorders

As shown in Table 2, two patients receiving aspirin had platelet count ↓; 12 patients receiving aspirin and six patients receiving DAPT had PT ↑; and 8 patients receiving aspirin and five patients receiving DAPT were confirmed as Fbg ↓. The mean AA% and ADP% of patients with PR was higher than those of patients with NPR (all *p* <0.05).

**Table 2:** The information of preoperative coagulation characteristics.

**Characteristics**	**Aspirin**		**Clopidogrel**		**DAPT**	
	**PR**	**NPR**	**PR**	**NPR**	**PR**	**NPR**
	***n*** **= 17**	***n*** **= 111**	***n*** **= 2**	***n*** **= 13**	***n*** **= 21**	***n*** **= 33**
Platelet count ↓^†^-no. (%)	1 (5.9%)	1 (0.9%)	0 (0.0%)	0 (0.0%)	0 (0.0%)	0 (0.0%)
APTT ↑^†^-no. (%)	0 (0.0%)	0 (0.0%)	0 (0.0%)	0 (0.0%)	0 (0.0%)	0 (0.0%)
PT ↑^†^-no. (%)	1 (5.9%)	11 (9.9%)	0 (0.0%)	0 (0.0%)	2 (9.5%)	4 (12.1%)
Fbg ↓^†^-no. (%)	3 (17.6%)	5 (4.5%)	0 (0.0%)	0 (0.0%)	5 (23.8%)	0 (0.0%)
CK-MA -no. (%)						
>70 mm	0 (0.0%)	7 (6.3%)	0 (0.0%)	2 (15.4%)	1 (4.8%)	5 (15.2%)
50~70 mm	11 (64.7%)	98 (88.3%)	2 (100%)	11 (84.6%)	15 (71.4%)	27 (81.8%)
<50 mm	6 (35.3%)	6 (5.4%)	0 (0.0%)	0 (0.0%)	5 (23.8%)	1 (3.0%)
AA% -median (IQR)	71.1 (51.8–77.9)	42.7 (27.4–54.4)	N/A	N/A	60.8 (53.8–70.4)	52.6 (36.1–58.6)
ADP% -median (IQR)	N/A	N/A	85.8 (79.3–92.3)	42.3 (31.4–52.1)	62.3 (48.7–64.3)	52.9 (44.0–74.8)

*N/A, not this data*.

† *Platelet count ↓, platelet count <50 ×10^9^; APTT ↑, APTT prolongs by ≥ 10 s; PT ↑, PT prolongs by ≥ 3 s; Fbg ↓, Fbg <1.5 g/l*.

*PR, postoperative rebleeding; NPR, no postoperative rebleeding; DAPT, dual antiplatelet treatment; APTT, activated partial thromboplastin time; PT, prothrombin time; Fbg, fibrinogen; CK-MA, citric acid kaolin-tracing maximum amplitude; AA%, the inhibition caused by aspirin; ADP%, the inhibition caused by clopidogrel*.

Of the included patients, 75 patients (38.1%) had the platelet dysfunction only, 13 (6.6%) had the coagulation factor dysfunction only, 13 (6.6%) had both dysfunctions, and 96 patients (48.7%) did not have any dysfunction (Figure 2C). The IRs of PR in patients with the coagulation factor dysfunction, the platelet dysfunction, both dysfunctions, and without any dysfunction were 0, 29.3, 69.2, and 9.4 per 100 persons, respectively (Figure 2D and Supplementary Table 2).

### Novel Coagulation Classification and PR

**Figure 3:**
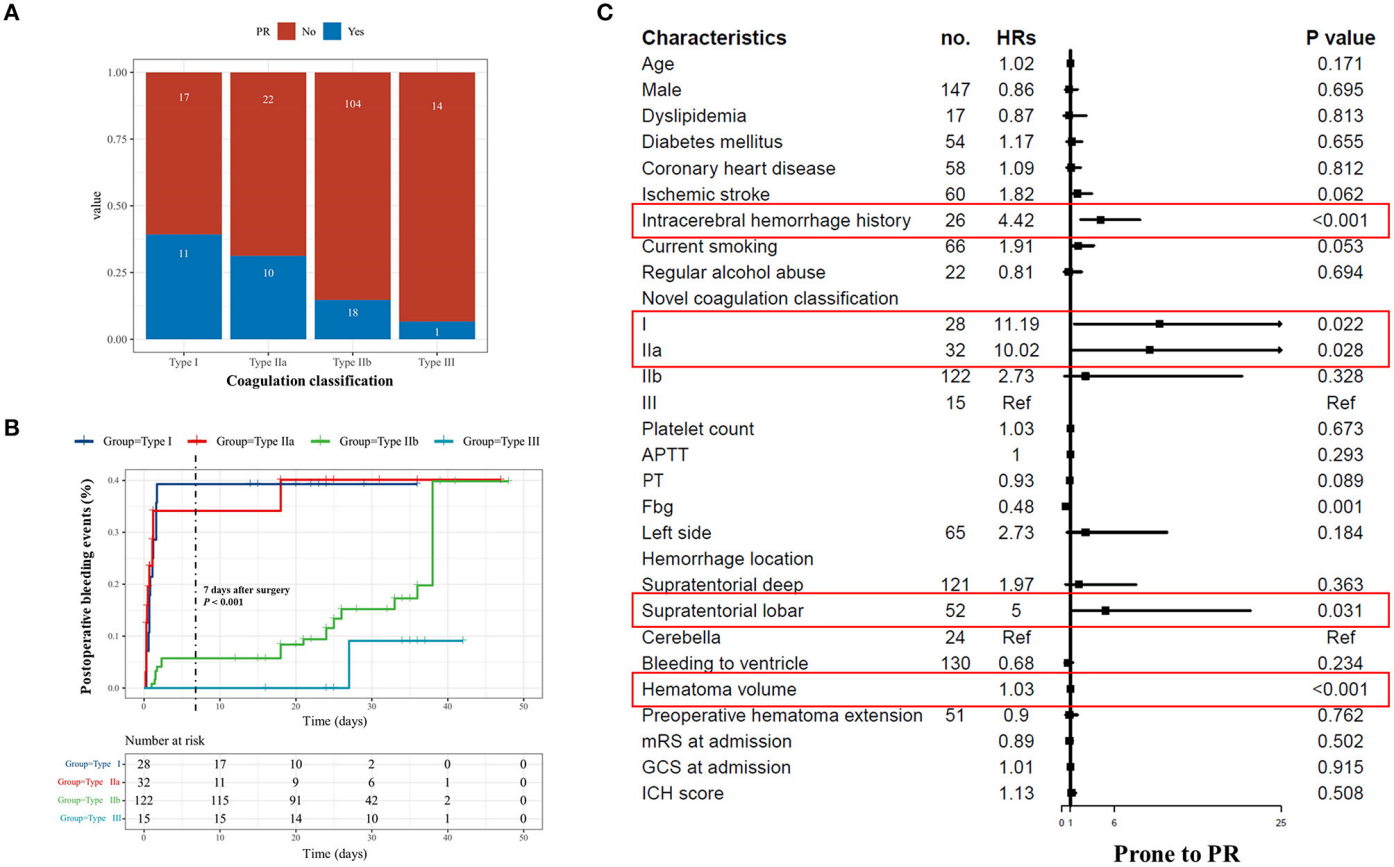
Coagulation classification and postoperative rebleeding. **(A)** The number of PR in each coagulation classification. **(B)** The survival curve PR based on our novel coagulation classification is presented. The Type I patients had the highest risk of PR, followed by the Types IIa, IIb, and III. Within 7 days after surgery, the incident rate of PR was significantly higher in the Type I and Type IIa patients compared with the Type IIb and Type III patients. **(C)** The forest plot presented the result of univariate Cox regression analysis. The red frames suggest the significant parameters, which are then input into multivariate analysis. PR, postoperative rebleeding; NPR, no postoperative rebleeding; HR, hazard ratio; APTT, activated partial thromboplastin time; Fbg, fibrinogen.

The IRs of PR were highest in the Type I patients (39.3 per 100 persons), followed by the Type IIa patients (31.3 per 100 persons). The Type III patients had the lowest IR of PR (6.7 per 100 persons) (Figure 3A). Survival analysis showed that the Type I patients had the highest risk of PR, followed by Types IIa, IIb, and III (Figure 3B). Within 7 days after surgery, the IR of PR was significantly higher in the Type I and Type IIa patients, compared with the Type IIb and Type III patients (*p* < 0.001).

The result of the Cox regression analyses is listed in Table 3. The univariate Cox regression indicates that intracerebral hemorrhage history (*p* < 0.001), APT prehemorrhage (*p* < 0.001), hemorrhage location (*p* = 0.005), hematoma volume (*p* < 0.001), Fbg (*p* = 0.001), CK-MA (*p* < 0.001), and novel coagulation classification (*p* < 0.001) as the risk factors for PR (Figure 3C and Supplementary Table 3). Since the novel coagulation classification had multicollinearity with APT prehemorrhage, CK-MA, and Fbg, we then input novel coagulation classification, intracerebral hemorrhage history, hemorrhage location, and hematoma volume into a multivariate regression model. Besides, the result demonstrated the large hematoma volume (HR: 1.02; 95% CI: 1.02–1.03; *P* < 0.001), Type I (HR: 9.72; 95% CI: 1.19–79.67; *p* = 0.034), and Type IIa (HR: 8.70; 95% CI: 1.09–69.61; *p* = 0.041) correlated with the highest risk of PR.

**Table 3:** Multivariate Cox regression analyses for risk factors related to the PR.

**Characteristics**	**HR**	**95% CI**	***P*** **value**
Novel coagulation classification			0.009
Type I	9.72	1.19–79.67	0.034
Type IIa	8.70	1.09–69.61	0.041
Type IIb	2.61	0.34–19.86	0.354
Type III	Ref	Ref	Ref
Hematoma volume	1.02	1.02–1.03	<0.001
Intracerebral hemorrhage history	0.77	0.33–1.78	0.534
Hemorrhage location			0.629
Supratentorial deep	0.66	0.21–2.04	0.466
Supratentorial lobar	0.55	0.16–1.87	0.340
Cerebella	Ref	Ref	Ref

*HR, hazard ratio; Ref, reference*.

The comparison of conventional coagulation assessment (only considering platelet count ↓, APTT ↑, PT ↑, or Fbg ↓) and novel coagulation classification is drawn in Table 4. The novel coagulation classification performed well in discriminating patients with PR from patients with NPR (*p* < 0.001), whereas the conventional coagulation classification failed to discriminate patients with PR from patients with NPR (*p* = 0.119). As shown in Supplementary Figure 2, the novel coagulation classification had a clinical utility and had a higher discriminating accuracy for PR compared with the conventional coagulation assessment (AUCs: 0.74 vs. 0.55).

**Table 4:** Comparison of conventional coagulation assessment and novel coagulation classification.

	**PR**	**NPR**	***P*** **value**
Conventional coagulation assessment^†^			
Disorder a, *n =* 29	9	20	0.119
No disorder, *n =* 168	31	137	
Novel coagulation classification			
Type I, *n =* 28	11	17	0.005
Type IIa, *n =* 32	10	22	
Type IIb, *n =* 122	18	104	
Type III, *n =* 15	1	14	

*The patients with any one of the platelet count <50 × 10^9^; APTT prolongs by ≥ 10 s, PT prolongs by ≥ 3 s, or Fbg <1.5 g/L were recognized as the conventional coagulation disorder.*

*PR, postoperative rebleeding; NPR, no postoperative rebleeding; IR, incident rate*.

## Discussion

Antiplatelet therapy could inhibit the platelet function, which cannot currently be screened by the regular preoperative coagulation examination. Our current study showed that platelet dysfunction is the main coagulation disorder in operated patients with APT-SICH and promoted a novel coagulation classification combining the platelet function and the coagulation factor function. The subsequent analyses here revealed that this novel coagulation classification could evaluate the risk of PR in operated patients with APT-SICH.

Emergency surgery was a safety consideration for patients with APT-SICH. Coagulation disorder caused by APT and the subsequent PR act as a barrier to the decision-making of emergency surgery, thereby commonly causing doctors to face a dilemma. This study found that PR occurred in 20.3% of operated patients with APT-SICH, which complied with our previous study (Wu et al., 2021b). Notably, for more than half of patients with APT-SICH, emergency surgery is still a considerable way to save their life.

Platelet dysfunction was the main coagulation disorder in patients with APT-SICH. This study found 38.1% of patients with platelet dysfunction but 6.6% of patients with coagulation factor dysfunction. Notably, the patients with platelet function had a higher risk of PR than the patients with coagulation factor dysfunction. This phenomenon also suggests the role of platelet function in maintaining coagulation, which could ensure the safety of surgery, especially in emergency conditions.

This study presented a novel coagulation classification, categorizing all patients with SAP-SICH into four types. The Type I patients had the highest risk of PR, whereas the Type III patients had the lowest risk. The process of hemostasis includes vasoconstriction, activation of platelet, and activation of coagulation factor system. Platelets critically impact coagulation amplification and propagation (Bezinover et al., 2018). Thus, ignoring the platelet function would underestimate the risk of PR in patients with APT-SICH. In practice, conventional coagulation assessment, only considering platelet count and coagulation factor function, was found with no ability to discriminate the patients with the highest risk of PR. In contrast, our novel coagulation classification performed well in discriminating the high-risk patients, and it outperformed the conventional coagulation assessment. For the Type I patients, there should be a severe dysfunction in platelet and coagulation factor. For this reason, the mentioned patients had the highest risk of PR. For the Type IIa patients, though the coagulation factor function and platelet function were normal, the platelet reserve capacity was quite low, which could not support the coagulation process once severe trauma or injury occurred (e.g., neurosurgery). Therefore, emergency surgery is not recommended for the Type I and Type IIa patients. As to the Type IIb and Type III patients, the coagulation factor function is normal, and the platelet function is normal or hyperfunction; therefore, they may have a lower risk of PR after surgery (IR of PR was 13.8 per 100 persons, which was approximately three times lower than that of the Type I and Type IIa patients). Notably, the Type IIb and Type III patients comprised more than 50% of all included patients. Thus, emergency surgery could be safe for considerable patients with APT-SICH. However, this study considered that the Type III patients may have aspirin resistance, so they could be more likely to have ischemic cerebrovascular or cardiovascular diseases postoperatively, which should be confirmed based on in-depth studies. In addition, because we did not validate the reliability of our classification using other independent cohorts, further studies were needed to confirm our conclusion.

Another interesting finding was the PR rate in different surgical methods. Due to better hemostasis during the craniectomy surgery, neurosurgeons prefer a craniectomy instead of minimally invasive surgery for patients with APT-SICH. However, our study revealed that more patients with APT-SICH receiving craniectomy had a PR, as compared with patients receiving minimal invasive surgery, even though there was no significance among different surgery methods. Compared with minimally invasive surgery, the craniectomy can cause more damage to patients. We considered that patients with APT-SICH with coagulation disorder cannot bear such a complex surgery, i.e., craniectomy. However, whether craniectomy is associated with a higher risk of PR needed further studies to investigate.

There were some limitations in this study. First, for preoperative symptoms (e.g., fever), some patients might take painkillers (e.g., salicylic acid antipyretics), which may also inhibit the platelet function. Although we excluded the patients who took aspirin effervescent tablets and aspirin-dl-lysine, there may be other medications that could also affect the platelet function. Second, the selection of surgery was largely up to the neurosurgeons. The doctor bias of the doctors may limit the conclusion here. However, since this study mainly focused on the relationship between the coagulation function and the postoperative bleeding complications, the conclusion here shows clinical utility. Third, only 15 patients administrated with clopidogrel were registered in this cohort. A small sample might not effectively represent the condition of this group. Fourth, this study did not consider intraoperative findings (e.g., prothrombin complex concentration injection, intraoperative bleeding, and hemostasis) and other factors that may be also related to PR, e.g., heparin administration. However, considering that most of PR occurred within a short time after emergency surgery, we thought that the coagulation and platelet function had a more significant effect on PR. Fifth, we excluded some patients who did not complete the TEG examination. These patients may also have a platelet dysfunction. The potential selection bias among the excluded patients may limit our conclusion. Despite the abovementioned existing problems, this study still provides a useful coagulation assessment tool for patients with APT-SICH and patients with APT requiring surgical treatment.

## Conclusion

Platelet dysfunction was found as the main coagulation disorder in operated patients with APT-SICH. Our novel coagulation classification, which outperformed the conventional coagulation assessment, could identify the preoperative coagulation disorder and is a valuable tool to preoperatively evaluate the risk of PR in patients with APT-SICH undergoing emergency surgery. For the Type I and Type IIa patients, surgery should be considered carefully because of the highest risk of PR.

## Data Availability Statement

The raw data supporting the conclusions of this article will be made available by the authors, without undue reservation.

## Ethics Statement

The studies involving human participants were reviewed and approved by Institutional Review Board of Beijing Tiantan Hospital. The patients/participants provided their written informed consent to participate in this study.

## Author Contributions

Conception and design were done by QL, JW, and XL. Acquisition of data was done by NW, JY, KW, SC, and JL. Analysis and interpretation of data were done by QL and YZ. Drafting the article was done by QL. Critically revising the article was done by YZ. Approving the final version of the manuscript on behalf of all authors and study supervision and correspondence was done by SW. Reviewing the submitted version of the manuscript was done by all authors. All authors contributed to the article and approved the submitted version.

## Funding

This study was supported by the National Natural Science Foundation of China (Grant Nos. 82071296, 81671129, and 81471210), National Key Research and Development Program of the 14th Five-Year Plan (Grant No. 2021YFC2501100), and TAIHU Top Talent Support Program for Top Medical Experts (Grant No. TH202109).

## Conflict of Interest

The authors declare that the research was conducted in the absence of any commercial or financial relationships that could be construed as a potential conflict of interest.

## Publisher's Note

All claims expressed in this article are solely those of the authors and do not necessarily represent those of their affiliated organizations, or those of the publisher, the editors and the reviewers. Any product that may be evaluated in this article, or claim that may be made by its manufacturer, is not guaranteed or endorsed by the publisher.

## Abbreviations

SICH: severe spontaneous intracerebral hemorrhage
APT: antiplatelet therapy
APT-SICH: patients with severe spontaneous intracerebral hemorrhage on antiplatelet therapy
PR: postoperative rebleeding
SAP-ICH study: surgical treatments for antiplatelet intracerebral hemorrhage study
DAPT: dual antiplatelet therapy
mRS: modified Rankin scale
GCS: Glasgow Coma Score
APTT: activated partial thromboplastin time
PT: prothrombin time
Fbg: fibrinogen
TEG: thrombelastography
CK-MA: citric acid kaolin tracing-maximum amplitude
AA%: the inhibition caused by aspirin
ADP%: the inhibition caused by clopidogrel.
