# Supplementary material for: A Novel Coagulation Classification and Postoperative Bleeding in Severe Spontaneous Intracerebral Hemorrhage Patients on Antiplatelet Therapy
Source: Front Aging Neurosci. 2022 Feb 16;14:793129. doi: 10.3389/fnagi.2022.793129 (PMC8888928; doi:10.3389/fnagi.2022.793129)
Supplement: Supplementary file 1 [file Data_Sheet_1.PDF]

Supplementary materials to *A novel coagulation classification and severe spontaneous intracerebral hemorrhage patients on antiplatelet therapy*

Supplementary tables

Online Table 1. The novel coagulation classification

	Platelet count	APTT	PT	Fbg	CK-MA	AA%/ADP%
Type I †	↓††	↑††	↑††	↓††	> 70 mm	
Type IIa	-	-	-	-	50~70 mm	↑††
Type IIb	-	-	-	-	50~70 mm	-
Type III	-	-	-	-	< 50 mm	

†, the patients with any one of the platelet count < 50×10^9, coagulation factor dysfunction (APTT prolongs by ≥ 10 seconds, PT prolongs by ≥ 3 seconds, Fbg < 1.5 g/l) or platelet hypofunction (CK-MA > 70 mm) were recognized as the Type I.

††, Platelet count ↓, platelet count < 50×10^9; APTT ↑, APTT prolongs by ≥ 10 seconds; PT ↑, PT prolongs by ≥ 3 seconds; Fbg ↓, Fbg < 1.5 g/l; AA%/ADP% ↑, AA% or ADP% > cutoff values.

Abbreviation: APTT, activated partial thromboplastin time; PT, prothrombin time; Fbg, fibrinogen; CK-MA, citric acid kaolin-tracing maximum amplitude; AA%, the inhibition caused by aspirin; ADP%, the inhibition caused by clopidogrel.

Online Table 2. The incident rate of PR in different preoperative coagulation disorder

Coagulation disorders	no.†	IR (95%CI) ††
Coagulation factor dysfunction	0	0.0
Platelet dysfunction	22	29.3 (18.8-39.9)
Both dysfunctions	9	69.2 (40.2-98.3)
No dysfunction	9	9.4 (3.4-15.3)

†, the number of PR patients.

††, the incident rate of PR per 100 persons.

Abbreviation: PR, postoperative rebleeding; NPR, no postoperative rebleeding; IR, incident rate.

Online Table 3. Univariate Cox regression analysis for factors associated with the PR

Characteristics	HR	95%CI	p value
Male	0.86	0.41-1.81	0.695
Age	1.02	0.99-1.05	0.171
Dyslipidemia	0.87	0.27-2.82	0.813
Diabetes mellitus	1.17	0.59-2.30	0.655
Coronary heart disease	1.09	0.55-2.14	0.812
Ischemic stroke	1.82	0.97-3.41	0.062
Intracerebral hemorrhage history	4.42	2.19-8.93	<0.001
Current smoker	1.91	0.99-3.68	0.053
Regular alcohol abuse	0.81	0.29-2.28	0.694
Antiplatelet therapy pre-hemorrhage			<0.001
Aspirin	0.27	0.14-0.52	<0.001
Clopidogrel	0.28	0.07-1.19	0.085
DAPT	Ref	Ref	Ref
Left side	2.73	0.62-12.01	0.184
Hemorrhage location			0.005
Supratentorial deep	1.97	0.46-8.45	0.363
Supratentorial lobar	5.00	1.16-21.48	0.031
Cerebella	Ref	Ref	Ref
Bleeding to ventricle	0.68	0.37-1.28	0.234
Hematoma volume	1.03	1.02-1.03	<0.001
Preoperative hematoma extension	0.90	0.44-1.83	0.762
mRS at admission	0.89	0.63-1.25	0.502
GCS at admission	1.01	0.92-1.10	0.915

ICH score	1.13	0.79-1.63	0.508
Surgery			0.098
Craniectomy	0.52	0.27-1.00	0.050
Endoscopic surgery	0.43	0.13-1.45	0.172
Minimally invasive surgery	Ref	Ref	Ref
Platelet count	1.03	0.99-1.07	0.673
APTT	1.00	0.99-1.00	0.293
PT	0.93	0.86-1.01	0.089
Fbg	0.48	0.31-0.75	0.001
CK-MA			<0.001
>70mm	27.72	3.49-220.06	0.002
50~70mm	3.67	0.50-27.04	0.202
<50mm	Ref	Ref	Ref
Novel coagulation classification			<0.001
I	11.19	1.43-87.86	0.022
IIa	10.02	1.28-78.68	0.028
IIb	2.73	0.36-20.54	0.328
III	Ref	Ref	Ref

Abbreviations: PR, postoperative rebleeding; mRS, modified Rankin scale; GCS, Glasgow coma score; APTT, activated partial thromboplastin time; Fbg, fibrinogen; HR, hazard ratio; CI, confidence interval; Ref, reference.

Supplementary figures

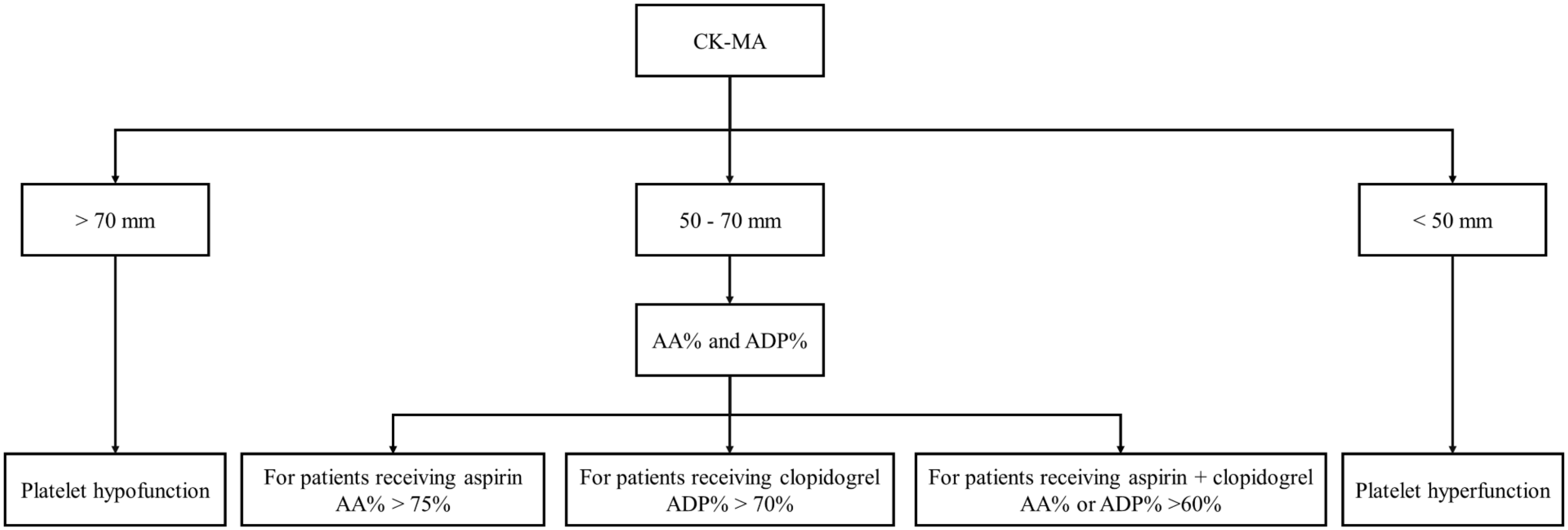

Online Figure 1. The mind mapping of thrombelastography analysis

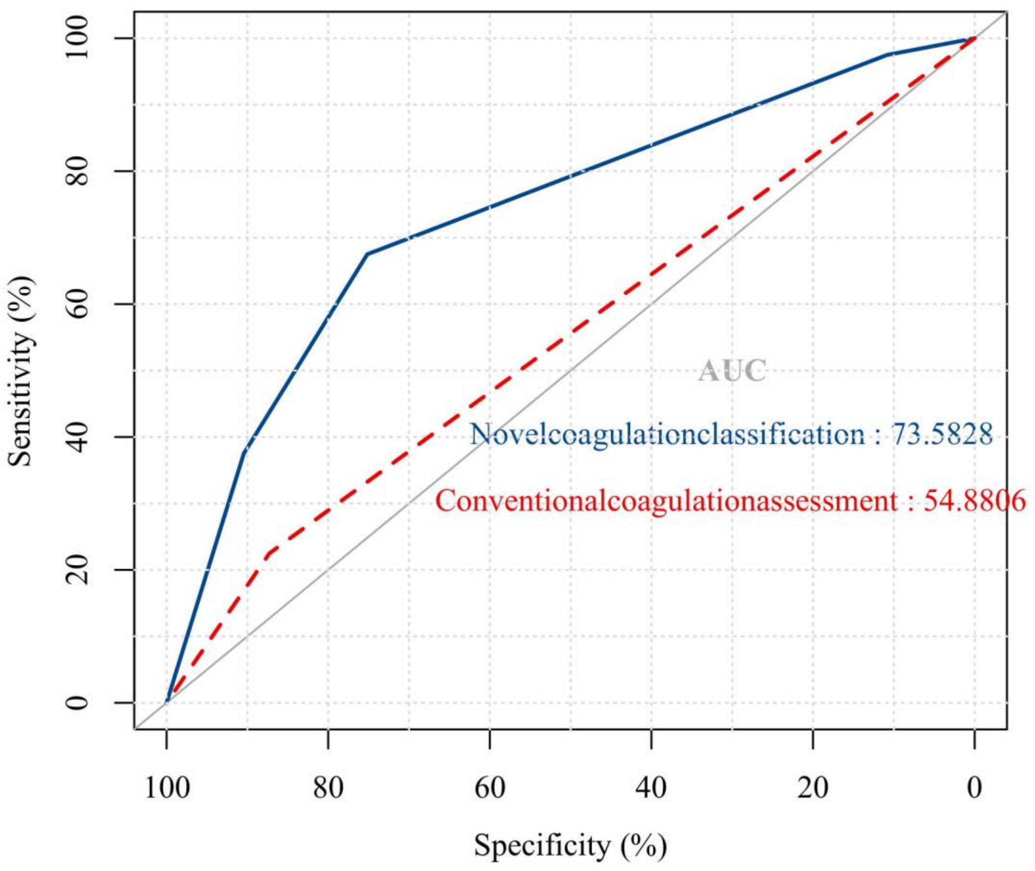

Online Figure 2. The comparison of the predictive accuracy of novel coagulation classification and conventional coagulation assessment for postoperative rebleeding
